# Clinical and molecular characteristics, antibiotic susceptibility, risk factors and predictors of mortality in carbapenem-resistant klebsiella pneumoniae bloodstream infections in Southern Sichuan, China: a 5-year multicenter study

**DOI:** 10.3389/fcimb.2026.1741170

**Published:** 2026-02-23

**Authors:** Wenchao Xie, Zixiang Luo, Chenghong Gu, yanzhi Li, Jie Deng, Yuling Yang, Yueshuai Wei, Yuan Jiang, Jinbo Liu, Zhangrui Zeng

**Affiliations:** 1 Department of Laboratory Medicine, The Affiliated Hospital, Southwest Medical University, Luzhou, China; 2 Sichuan Province Engineering Technology Research Center of Clinical Diseases Molecular Diagnosis, Luzhou, China; 3 Clinical Diseases Molecular Diagnosis Key Laboratory of LuZhou, Luzhou, China; 4 Department of Laboratory Medicine, Zigong Fourth People’s Hospital, Zigong, China; 5 Department of Laboratory Medicine, the Second People’s Hospital of Neijiang, Neijiang, China

**Keywords:** bloodstream infections, carbapenem-resistant Klebsiella pneumoniae, clinical and molecular characteristics, epidemiology, risk factors

## Abstract

**Background:**

The World Health Organization (WHO) has listed carbapenem-resistant *Klebsiella pneumoniae* (CRKP) as one of the key pathogens that require the highest priority for containment. However, there is still a lack of systematic reports on bloodstream infections(BSI) caused by CRKP in the southern Sichuan of China. This study aimed to retrospectively analyze the clinical characteristics, risk factors, mortality predictors, and molecular epidemiological features of CRKP-BSI strains in patients from three teaching hospitals in southern Sichuan from 2020 to 2024.

**Methods:**

Clinical and laboratory electronic medical records of patients with KP-BSI were collected retrospectively. T-tests, Mann-Whitney U tests, logistic regression, and survival curve analyses were used to assess risk factors and mortality predictors. Strains were re-identified and verified by matrix-assisted laser desorption/ionization time-of-flight mass spectrometry (MALDI-TOF MS). Antimicrobial susceptibility testing was performed using the microbroth dilution method. Molecular typing was based on multilocus sequence typing (MLST) and pulsed-field gel electrophoresis (PFGE). Capsular serotypes, virulence genes, and virulence phenotypes were detected using polymerase chain reaction (PCR), sequencing, and serum resistance assays.

**Results:**

A total of 430 cases meeting the criteria were included, of which 66 were CRKP BSI cases. The highest isolation rate of CRKP BSI was in the intensive care unit (ICU, 28.8%). The resistance rate of CRKP to carbapenems was above 92.2%, and the 28-day mortality rate of CRKP-BSI patients was 43.9%. Multivariate analysis showed that pulmonary infection (*P* = 0.021), ICU stay >3 days (*P* = 0.029), various invasive catheters (*P* < 0.001), and combination with three or more antibiotics (*P* < 0.001) were independent risk factors for CRKP-BSI. Mechanical ventilation (*P* = 0.014) and receipt of corticosteroids >7 days (*P* = 0.028) were independent risk factors for patient mortality, with the latter also significantly associated with shortened survival time. Molecular epidemiology revealed that ST11 was the dominant clone (43.33%), with three new sequence types identified: ST6845, ST6846, and ST6847. The most common carbapenemase gene was *blaKPC-2* (60.0%) and *blaNDM-5* (20.0%), and the most common virulence gene was *ybtA* (76.7%). The outer membrane porin genes *ompk35* and *ompk36* were detected in 96.7% and 83.3% of the strains, respectively. The positivity rate for biofilm formation was 83.3%, while the positivity rate for the hypermucoviscous phenotype (string test) was 6.67%, and the serum resistance phenotype was detected in 36.7% of cases.

**Conclusion:**

Carbapenem-resistant Klebsiella pneumoniae (CRKP) in southern Sichuan, China, is characterized by high resistance rates, the continuous emergence of novel clones, and elevated patient mortality. These findings underscore the urgent need for enhanced surveillance that integrates clinical and molecular epidemiology with targeted interventions. This study provides a robust scientific foundation for optimizing the clinical management of CRKP bloodstream infections. This study provides a scientific basis for optimizing clinical management strategies for CRKP-BSI.

## Introduction

*Klebsiella pneumoniae* (KP) ranks among the most prevalent Gram-negative opportunistic pathogens encountered in clinical practice. It is a major contributor to hospital-acquired infections but also capable of causing severe invasive infections, including sepsis, septicemia, pneumonia, urinary tract infections, surgical site infections, and soft tissue infections (Choby et al., 2020). Epidemiological surveillance data reveal that the detection rate of KP in clinical isolates in China is second only to that of *Escherichia coli* and continues to rise (Yan et al., 2024). Notably, the increasing selective pressure exerted by antimicrobial agents, coupled with the horizontal transfer of resistance genes, has exacerbated the epidemiological situation of carbapenem-resistant *K. pneumoniae* (CRKP) (Han et al., 2025; Spadar et al., 2023). Surveillance data in China show that the resistance rates of KP isolated from all samples to imipenem and meropenem have significantly increased from 3.0% and 2.9% in 2005 to 24.8% and 26.0% in 2023 (Yan et al., 2024), respectively. This alarming trend is consistent with the global antimicrobial resistance situation. Data from the European Antimicrobial Resistance Surveillance Network (EARS-Net) indicate that 25% of EU countries report CRKP detection rates exceeding 10%, with particularly severe resistance situations in Italy (29.5%), Romania (48.3%), and Greece (66.3%) (Onorato et al., 2022).

In clinical practice in China, KP has emerged as the second most common Gram-negative pathogen causing bloodstream infections (BSI). The latest data from the CHINET surveillance network show that the resistance rates of *K. pneumoniae* bloodstream infections (KP-BSI) isolates to imipenem and meropenem increased from 19.9% and 21.4% in 2015 to 25.7% and 26.6% in 2021, respectively (Zhong et al., 2024). Some tertiary hospitals in North China have even reported that the proportion of CRKP in KP-BSI reached 42% (Li et al., 2020). More alarmingly, the mortality rate of patients with carbapenem-resistant *K. pneumoniae* bloodstream infections(CRKP-BSI) can be as high as 42-81% (Cheng et al., 2024), posing a significant challenge to clinical treatment. Therefore, a systematic investigation of the risk factors and clinical epidemiological characteristics of CRKP-BSI is of great public health significance for developing effective infection control strategies.

However, there is currently a lack of multicenter and systematic studies on CRKP-BSI in the southern Sichuan region, and molecular epidemiological data are also unavailable. Systematically elucidating the risk factors, clinical characteristics, and molecular features of the pathogens associated with CRKP-BSI in this area is an urgent need for formulating precise infection control strategies and holds significant public health importance. This study aims to characterize the clinical epidemiological profile of CRKP-BSI, identify risk factors for infection and mortality, and profile the molecular and virulence characteristics of the isolates, thereby providing a robust evidence base for regionalized and targeted prevention and control measures.

## Methods

### Study design

We conducted a retrospective observational study of electronic laboratory records of CRKP-BSI patients from the Affiliated Hospital of Southwest Medical University (AHSWMU; Luzhou, China), Zigong Fourth People’s Hospital (ZGFPH; Zigong, China) and the Second People’s Hospital of Neijiang (SPHNJ; Neijiang, China) from January 2020 to December 2024. The AHSWMU is a 3200-bed tertiary care teaching hospital with 43 wards and approximately 130,000 annual admissions, the ZGFPH is a 1600-bed tertiary care teaching hospital with 32 wards and approximately 70,000 annual admissions, and the SPHNJ is a 1500-bed tertiary care teaching hospital with 38 wards and approximately 45,000 annual admissions. During this period, AHSWMU isolated and collected 426 cases of KP, ZGFPH isolated and collected 266 cases of KP, and SPHNJ isolated and collected 81 cases of KP. Among these cases, CRKP strains were detected in 57, 17, and 2 patients, respectively, yielding a total of 76 CRKP cases.

### Data collection

The data were collected from patients with KP-BSI admitted to the AHSWMU, ZGFPH and SPHNJ from January 2020 to December 2024. All data were collected from electronic medical records. The following data were retrospectively collected from all adult patients: demographic characteristics, underlying comorbidities, and clinical outcomes. Data on the following risk factors associated with CRKP-BSI were also collected: septic shock, co-infection with other bacteria/fungi, receipt of corticosteroids >7 days, ICU stay > 3 days, mechanical ventilation, central venous catheter (CVC) or peripherally inserted central catheter (PICC), parenteral nutrition, malnutrition, renal dialysis, use of antifungal agents, surgery, various invasive catheters, combination of three or more antibiotics, use of carbapenems before positive blood culture, use of tigecycline, procalcitonin (PCT) > 100 ng/ml. The study protocol was approved by the Clinical Research Ethics Committee of the Affiliated Hospital of Southwest Medical University (Project No. KY2022267). The need for informed consent was waived by the Clinical Research Ethics Committee of the Affiliated Hospital of Southwest Medical University. All experiments were performed according to the study protocol in three hospitals.

### Inclusion/exclusion criteria

The diagnostic criteria of BSI were based on the Expert Consensus on Clinical Laboratory Strategies for Bloodstream Infection, the expert consensus issued by the Shanghai Society of Critical Care Medicine of Shanghai Medical Association and the Clinical Microbiology Division of Shanghai Society of Microbiology (Clinical Microbiology Division of Shanghai Society of Microbiology et al., 2022). These criteria are also in accordance with the diagnostic criteria for bloodstream infections issued by the Centers for Disease Control and Prevention/National Healthcare Safety Network (CDC/NHSN) (Martinez and Wolk., 2016; Timsit et al., 2020). Bloodstream infection refers to the presence of pathogenic microorganisms in a patient’s bloodstream, with or without accompanying signs and symptoms of infection. Inclusion Criteria: Patients aged ≥16 years; Blood culture-confirmed KP-BSI that meets the diagnostic criteria for bloodstream infection; Hospitalized patients with complete records in the electronic medical record system, with traceable key clinical diagnostic and therapeutic data; Infection cases confirmed by antimicrobial susceptibility testing to be CRKP strains (judged according to the CLSI breakpoint standards of the respective year). Exclusion Criteria: Patients under 16 years of age; Short-term observation cases with a hospital stay of less than 48 hours; Patients diagnosed with CRKP-BSI and treated with targeted antimicrobial therapy before admission(Defined as patients who, before transfer to the three study hospitals, had been diagnosed with CRKP-BSI by blood culture and antimicrobial susceptibility testing at an external facility and had already received targeted therapy.); Recurrent infections: For patients with multiple isolations of CRKP strains, only the first infection event and its corresponding clinical data are included; Cases with missing or incomplete records of key clinical medical information.

### Microorganism identification and susceptibility testing

According to the manufacturer’s instructions, blood(10ml) was inoculated into both aerobic and anaerobic BacT/AlerT 3D vials (bioMérieux, France). All positive cultures were manually sampled and inoculated into Columbia blood agar plates and Chocolate blood agar plates to ensure viability and purity. The identification of all species was confirmed by a Vitek-2 system (bioMérieux, Marcy L’Etoile, France) at SPHNJ and Microflex LT (Bruker Diagnostics Inc., USA) matrix-assisted laser-desorption/ionization time-of-flight mass spectrometry system at AHSWMU and ZGFPH.

Antimicrobial susceptibility tests(AST) was performed using the VITEK 2 Compact System according to the manufacturer’s instructions, and results were interpreted using CLSI breakpoints; where necessary, MICs were confirmed by manual broth microdilution, and the results were interpreted according to Clinical Laboratory and Standards Institute (CLSI) breakpoints for the respective years (CLSI document M100-S20-23. Wayne, PA: CLSI, 2020-2023). CRKP was determined according to the CLSI guidelines. The control bacterial strains were *E. coli* ATCC 25922 and *K. pneumoniae* ATCC 13883 and 700603.

### Pulsed-field gel electrophoresis, multilocus sequence typing and construction of phylogenetic tree

PFGE was performed on *K. pneumoniae* isolates. Total DNA was extracted and digested with the XbaI restriction enzyme following the procedures described in the PulseNet International protocols for Salmonella. Electrophoresis was conducted using the CHEF-DR III PFGE system (BioRad, USA) under the following conditions: molecular weight setting of 30 kb-900 kb; voltage at 6 volts; angle at 120°; initial switch time 2.16s; final switch time 63.8s; and electrophoresis time of 19 hours. The result was performed for molecular typing according to the method described by Tenover et al., (1995).

MLST analysis was conducted by PCR amplifying seven housekeeping genes of *K. pneumoniae* (*gapA, infB, mdh, pgi, phoE, rpoB*, and *tonB*), followed by sequencing of the amplified fragments. The sequencing results were used to assign sequence types (STs) via the *K. pneumoniae* MLST website (http://www.pasteur.fr/recherche/genopole/PF8/mlst/Kpneumoniae).

The phylogenetic tree was constructed using the Neighbor-Joining method in MEGA 7. The analysis was performed based on the multiple sequence alignment of housekeeping gene sequences from Klebsiella pneumoniae. Evolutionary distances were computed using the p-distance model. Sites containing gaps and missing data were handled by the Partial deletion option, with a site coverage cutoff set at 50%. The robustness of the phylogenetic tree topology was evaluated by Bootstrap analysis with 1000 replicates. Branches with support values below 50% in the Bootstrap replicates were collapsed. The final phylogenetic tree was visualized and annotated using the Tree Explorer module within MEGA 7.

### Modified carbapenem inactivation method for detecting carbapenemases and PCR for detecting resistance genes, capsule genes, and virulence genes

The modified carbapenem inactivation method (mCIM) and the ethylenediaminetetraacetic acid (EDTA)-modified carbapenem inactivation method (eCIM) were used for the preliminary screening of carbapenemase production. A simplified procedure is outlined as follows: Fresh bacterial colonies were suspended and adjusted to a 0.5 McFarland standard in Tryptic Soy Broth (TSB). For the mCIM test, 2 mL of this suspension was aliquoted into a tube. For the eCIM test, 20 µL of 0.5 M EDTA (pH 8.0) was added to another 2 mL aliquot of the suspension. A 10 µg meropenem disk was immersed into each tube, followed by incubation at 35 °C for 4 hours. Concurrently, Mueller-Hinton agar plates were inoculated with a lawn of E. coli ATCC 25922 (0.5 McFarland standard). After incubation, the disks were removed and placed onto the surfaces of the seeded agar plates. All plates were then incubated at 35 °C for 18–24 hours. The inhibition zones were measured after incubation. An isolate was considered carbapenemase-positive by mCIM if the inhibition zone diameter was ≤15 mm or if distinct colonies were observed within a 16–18 mm zone. For mCIM-positive isolates, a difference in zone diameter where the eCIM result was ≥5 mm larger than the mCIM result identified the strain as MBL-producing; a difference of <5 mm indicated the production of a serine carbapenemase. Protocols and results were interpreted using the Clinical and Laboratory Standards Institute (CLSI) guidelines (2023-M100).

Bacterial DNA extraction was performed using the boiling method. Specifically, five single purified colonies were selected, resuspended in a PE tube containing 500 µL of sterile distilled water, and boiled at 100°Cfor 10 minutes. Subsequently, the mixture was centrifuged at 12,000 rpm for 10 minutes, and the supernatant was collected and stored at-80°Cfor subsequent use. PCR was used to amplify the following genes: carbapenemase genes (*bla_OXA-48_, bla_VIM_, bla_NDM_, bla_IMP_, and bla_KPC_*), ESBL genes (*bla_CTX-M-1_, bla_CTX-M-9_, bla_SHV_, and bla_TEM_*), outer membrane porin genes (*ompk35*, *ompk36*), capsule genes(*wzi*), common virulence genes (*rmpA, rmpA2, peg-344, aerobactin, iucA, iroN/B, YbtA, iutA*). PCR was performed in a 25-μL volume consisting of initial denaturation at 94 °C for 5 min, followed by 30 cycles of 94 °C for 30 s, annealing for 30 s (primer-specific Tm values are listed in **Supplementary Table 1**), and extension at 72 °C for 40 s, with a final extension at 72 °C for 6 min. The negative control was performed by substituting the template with sterile deionized water; positive amplification products were confirmed by sequencing. The primers used were designed as previously described (Wang et al., 2018; Zhang et al., 2014; Liao et al., 2022; Wang et al., 2021). All primers used in this study are listed in **Supplementary Table 1**. The PCR-positive products were sent to Sangon Biotech (Shanghai) Co., Ltd. for sequencing. The amplified sequences were analyzed and compared to the BLAST database(http://www.ncbi.nlm.nih.gov/BLAST/).

### Biofilm formation, Serum resistance assays, String test and Galleria mellonella infection modelstring test

As previously detailed, the capacity for biofilm formation was assessed via crystal violet staining [28]. The experimental setup included three biological replicates, with absorbance measured at 570 nm utilizing a microplate reader (PerkinElmer, USA). The biofilm-forming ability of the isolates was categorized into four levels: non-adherent (0, OD ≤ ODC), weakly positive (+, ODC < OD ≤ 2×ODC), moderately positive (++, 2×ODC < OD ≤ 4×ODC), and strongly positive (+++, OD > 4×ODC).

The virulence potential of these isolates was evaluated through serum resistance assays. Fresh colonies were selected from blood agar plates, and the concentration of the bacterial suspension was adjusted to 0.5 McFarland standards and subsequently diluted to 1×10^6^ CFU/mL with sterile Luria-Bertani (LB) broth. Then, 25 µL of the bacterial solution was mixed with 75 µL of serum obtained from healthy individuals. The mixture was incubated at 37 °C with shaking, and samples were taken at 0, 1, 2, and 3 hours for dilution and plating on LB agar for viable cell counts, denoted as N0, N1, N2, and N3, respectively. The data obtained were used to classify the tested strains into six grades: Grade 1: N1/N0 and N2/N0 < 10%, N3/N0 < 0.1%; Grade 2: N1/N0 and N2/N0 < 10%-100%, N3/N0 < 10%; Grade 3: N1/N0 > 100%, N2/N0 and N3/N0 < 100%; Grade 4: N1/N0 and N2/N0 > 100%, N3/N0 < 100%; Grade 5: N1/N0 and N2/N0 > 100%, N3/N2 < 100%; Grade 6: N1/N0 and N2/N1 > 100%, N3/N2 > 100%. Grades 1 and 2 were defined as high sensitivity, Grades 3 and 4 as moderate sensitivity, and Grades 5 and 6 as serum-resistant.

The string test was conducted as follows: Bacteria were inoculated onto Columbia blood agar plates using the quadrant streak method and incubated at 37°Cfor 18–24 hours. A disposable inoculation loop was used to select a single colony from the blood agar plate, which was then gently pulled upward repeatedly(more than three times).If the length of the mucoid string exceeded 5 mm, the string test was deemed positive, indicating a high mucoid phenotype of the strain; otherwise, the test was considered negative.

Construction of the Galleria mellonella Larval (GML) Infection Model: The method was modified according to the references (McLaughlin et al., 2014). The bacterial suspension was cultured in LB broth at 37°C overnight, then centrifuged and washed twice with PBS, and resuspended in 0.9% sterile saline to adjust the concentration to 1×10^6^ CFU/mL. Healthy and uniformly sized *Galleria mellonella* larvae without black spots on the surface were selected. The bacterial suspension (10 µL) was injected into the larvae via the second last right proleg. Each group consisted of 10 larvae. The negative control group was injected with an equal volume of sterile saline. After injection, the larvae were kept in the dark at 37°C with food and observed every 12 hours for 72 hours to record the number of surviving larvae. The experiment was considered valid if all the larvae in the negative control group survived. We assessed the bacterial virulence based on the specific temporal differences in the survival curves, using the highly virulent reference strain NTUH-K2044 and the low-virulent reference strain ATCC 700603 as references for high and low virulence, respectively. Strains with survival times identical to NTUH-K2044 were classified as highly virulent; those with survival times identical to ATCC 700603 were classified as low virulent; and those with survival times intermediate between NTUH-K2044 and ATCC 700603 were classified as moderately virulent. All experiments were independently repeated three times.

### Statistical analyses

Data analysis was performed using IBM SPSS Statistics version 27 for Windows (SPSS Inc., Chicago, IL, USA) and GraphPad Prism version 10(GraphPad Software, La Jolla, CA, USA). Categorical variables were compared using chi-square tests or Fisher’s exact tests, as appropriate. Continuous variables were analyzed using Student’s t-tests or Mann-Whitney U tests, depending on the distribution of the data. Multivariable logistic regression analysis was conducted to identify independent predictors of persistent colonization (PC)and 28-day hospital mortality. Odds ratios (ORs) and 95%confidence intervals (CIs)were calculated for each predictor. The multivariable logistic regression model based on biological plausibility included variables with a P value<0.1 in univariate analyses. Additionally, we employed Kaplan-Meier survival analysis with the log-rank test to evaluate the survival time of CRKP patients and generate survival curves; concurrently, we applied Cox proportional hazards regression to analyze factors influencing patient survival time. The handling of missing data: Variables with less than 5% missing data were analyzed using complete-case analysis; variables with 5%–20% missing values were handled by multiple imputation (m=5, PMM method); variables with more than 20% missing values were excluded from the multivariable models. Multicollinearity among covariates was evaluated by variance inflation factor (VIF); variables with VIF > 10 were either removed or combined. All key variables (ICU stay, mechanical ventilation, invasive procedures, septic shock) had VIF values < 3, indicating no significant multicollinearity. Statistical significance was determined using two-tailed tests, with *P* < 0.05 considered significant.

## Results

### Epidemiological investigation

**Figure 1:**
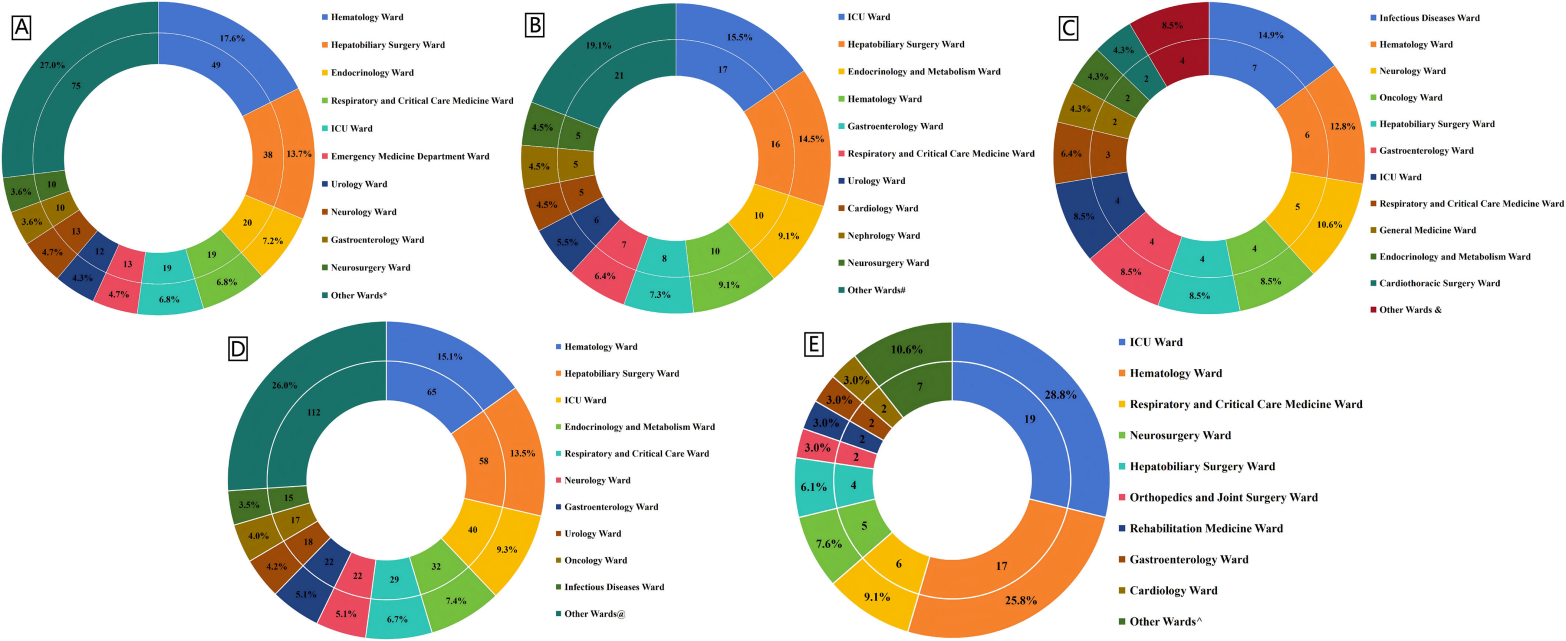
Distribution of KPBSI and CRKPBSI patients across different hospitals and wards. **(A)** Distribution of KPBSI patients across different wards in the Affiliated Hospital of Southwest Medical University, *: The data of the other wards are presented in **Supplementary Table 2**. **(B)** Distribution of KPBSI patients across different wards in Zigong Fourth People’s Hospital. #: The data of the other wards are presented in **Supplementary Table 2**. **(C)** Distribution of KPBSI patients across different wards in the Second People’s Hospital of Neijiang. &: The data of the other wards are presented in **Supplementary Table 2**. **(D)** Distribution of total KPBSI patients across different wards in the three hospitals. @:The data of the other wards are presented in **Supplementary Table 2**. **(E)** Distribution of total CRKPBSI patients across different wards in southern Sichuan, China. ^: The data of the other wards are presented in **Supplementary Table 2**.

Although the initial electronic screening identified a total of 773 cases of KP-BSI and 76 cases of CRKP-BSI, after rigorous selection according to the exclusion criteria, a total of 430 cases of KP-BSI that met the inclusion criteria were collected from three hospitals, including 278 cases from the AHSWMU,110 cases from ZFPH, and 51 cases from NSPH. Among the three hospitals, the number of CRKP-BSI patients detected was 47 cases, 17 cases, and 2 cases, respectively, totaling 66 cases (Another 10 cases were excluded: 6 had been diagnosed with CRKP-BSI outside the hospital and had already received targeted treatment, and 4 were excluded due to being under 16 years of age.) At the AHSWMU, the top five departments with the highest isolation rates of *K. pneumoniae* were Hematology Ward (49 cases, 17.6%), Hepatobiliary Surgery Ward (38cases, 13.7%), Endocrinology Ward (20 cases, 7.2%), Respiratory and Critical Care Medicine Ward (19 cases, 6.8%), and ICU Ward (19 cases, 6.8%). At ZFPH, the top five wards with the highest isolation rates were ICU Ward (17 cases, 16.2%), Hepatobiliary Surgery Ward (16 cases, 15.2%), Endocrinology and Metabolism Ward (10 cases, 9.5%), Hematology Ward (10 cases, 9.5%), and Gastroenterology Ward (8 cases, 7.6%). At NSPH, the top five wards with the highest isolation rates were the Infectious Diseases Ward (7 cases, 14.9%), Hematology Ward (6 cases, 12.8%), Neurology Ward (5 cases, 10.6%), Oncology Ward (4 cases, 8.5%), and Hepatobiliary Surgery Ward (4 cases, 8.5%). In the three cities in southern Sichuan, the top five wards with the highest total isolation rates of KP-BSI were Hematology Ward (65 cases, 15.1%), Hepatobiliary Surgery Ward (58 cases, 13.5%), ICU Ward (40 cases, 9.3%), Endocrinology and Metabolism Ward (32 cases, 7.4%), and Respiratory and Critical Care Medicine Ward (29 cases, 6.7%). The top five wards with the highest CRKP BSI were ICU Ward (19 cases, 28.8%), Hematology Ward (17 cases, 25.5%), Respiratory and Critical Care Medicine Ward (6 cases, 9.1%), Neurosurgery Ward (5 cases, 7.6%), and Hepatobiliary Surgery Ward (4 cases, 6.1%). For more detailed information, please refer to **Figure 1**.

### Antibiotic resistances

**Figure 2:**
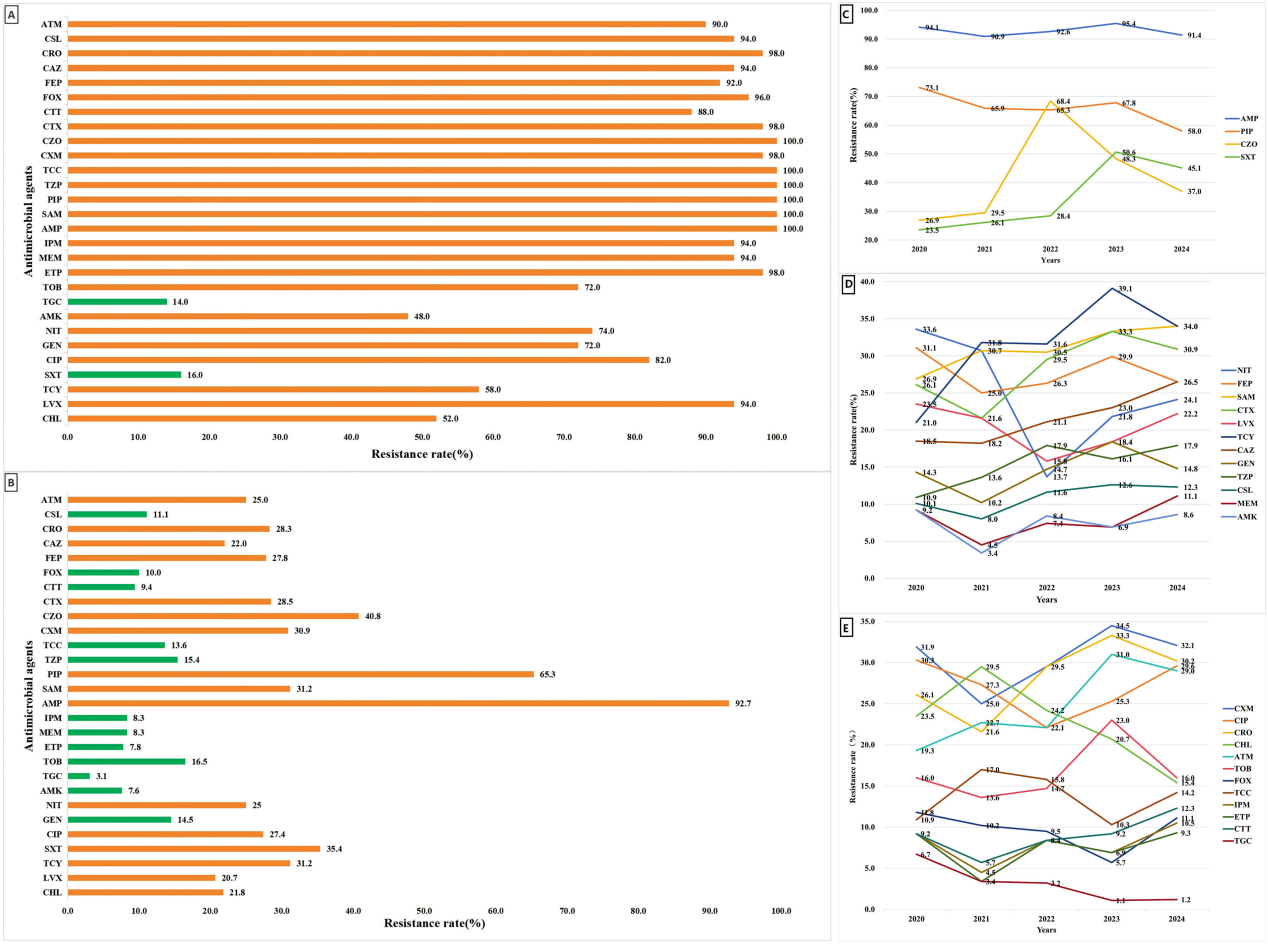
Antimicrobial susceptibility and dynamic trends of KP and CRKP strains. **(A)** Antimicrobial susceptibility profile of Klebsiella pneumoniae strains in southern Sichuan, China. **(B)** Antimicrobial susceptibility profile of carbapenem-resistant Klebsiella pneumoniae strains in southern Sichuan, China. **(C–E)** Dynamic trends in antimicrobial susceptibility of Klebsiella pneumoniae strains in southern Sichuan, China.

*In vitro* antimicrobial susceptibility testing, among the all of CRKP strains isolated from bloodstream infections, 96.1% exhibited resistance to ertapenem. The resistance rates to imipenem and meropenem were 92.2% and 92.2%, respectively. Over a five-year period, the resistance rates of *K. pneumoniae* isolates from bloodstream infections to imipenem, meropenem, and ertapenem were 8.3%, 8.3% and 7.8%, respectively. The resistance trends for these three carbapenem antibiotics remained relatively stable throughout this period. In contrast, the resistance rates to aztreonam and ceftazidime demonstrated a significant increase. The five-year resistance rates and trends for other antibiotics are depicted in **Figure 2**.

### Clinical baseline characteristics and risk factors for infection in KP-BSI patients

**Figure 3:**
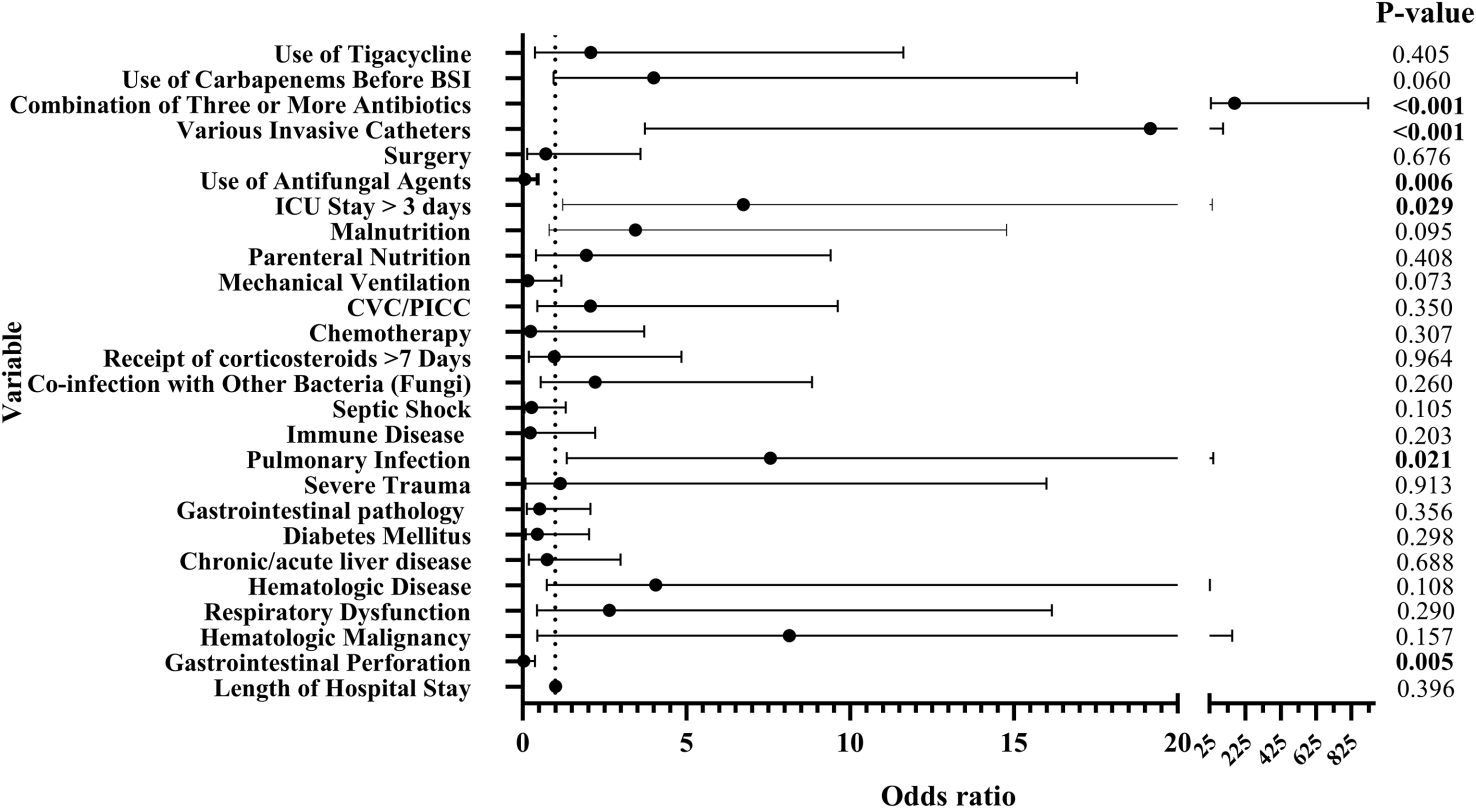
Factors associated with the formation of CRKP-BSI by multivariate analysis. Length of hospital stay(95% CI: 1.009–1.031), Gastrointestinal Perforation(95% CI: 0.040–0.381), Use of Antifungal Agents (95% CI: 0.064–0.458). Combination of Three or More Antibiotics:OR= 163.39, the precision of this estimate is low and should be interpreted with care.

The cohort characteristics are described in **Table 1**. Among the included patients (median age, 59 years), 55.3% were male. The average length of hospital stay was 26.5 days. The 28-day mortality rate among patients with bloodstream infections caused by CRKP was 12.6%, whereas the 28-day mortality rate among patients with CRKP-BSI was as high as 43.9%.The underlying diseases of the patients were most frequently pulmonary infection (191 cases,44.4%), followed by chronic/acute renal failure (169 cases,39.3%), diabetes mellitus (156 cases,36.3%), cardiovascular disease (152 cases,35.3%), chronic/acute liver disease (132 cases,30.7%), and gastrointestinal pathology (132 cases,30.7%). A comparative analysis of the underlying diseases in patients with CRKP-BSI and Carbapenem-susceptible *K. pneumoniae* bloodstream infections(CSKP-BSI) demonstrated that the incidence of hematologic malignancy, respiratory dysfunction, hematologic disease, chronic/acute liver disease, severe trauma, pulmonary infection, and immune disease was significantly higher in CRKP patients than in CSKP patients (*P* < 0.05). In terms of risk factors, except for renal dialysis and procalcitonin levels exceeding 100 ng/mL, all other risk factors were significantly more prevalent in patients with CRKP-BSI than in those with CSKP-BSI(*P* < 0.05). The detailed results are shown in **Table 2**. Multivariate logistic regression analysis identified pulmonary infection (odds ratio [OR], 7.57; 95% confidence interval [CI], 1.35–42.40; *P* = 0.021), ICU stay exceeding 3 days (OR, 6.74; 95% CI, 1.22–37.23; *P* = 0.029), use of various invasive catheters (OR, 19.17; 95% CI, 3.73–98.45; *P* < 0.001), and the combination of three or more antibiotics (OR, 163.39; 95% CI, 29.03–919.80; *P* < 0.001) as independent risk factors for CRKP-BSI infection (**Figure 3**).

**Table 1:** The difference of characteristics, underlying comorbidities and risk factors between CRKP and CSKP BSI inpatients.

Variable	KP-BSI (n=430)	CRKP-BSI (n=66)	CSKP-BSI (n=364)	*P-*value
Gender(Male:Female)	238:192	39:27	199:165	0.161
Age (median, range,years)	59(16~95)	53(16~95)	60(17~95)	0.506
Length of hospital stay (M:SD) (days)	26.5(29.8)	35.1(25.3)	24.9(33.3)	0.011
Underlying comorbidities (n,%)				
Gastrointestinal Perforation	61(14.2)	3(4.5)	58(15.9)	0.015
Solid Tumor	48(11.2)	4(6.1)	44(12.1)	0.153
Hematologic Malignancy	32(7.4)	17(25.8)	15(4.1)	<0.001
Respiratory Dysfunction^a^	107(24.9)	34(51.5)	73(20.1)	<0.001
Hematologic Disease	86(20)	37(56.1)	49(13.5)	<0.001
Chronic/acute liver disease	132(30.7)	33(50)	99(27.2)	<0.001
Diabetes Mellitus	156(36.3)	13(19.7)	143(39.3)	0.002
Chronic/acute renal failure^b^	169(39.3)	25(37.9)	144(39.6)	0.797
Cardiovascular Disease	152(35.3)	21(31.8)	131(36)	0.514
Gastrointestinal pathology^c^	132(30.7)	27(40.9)	105(28.8)	0.051
Neurological Disease	110(25.6)	15(22.7)	95(26.1)	0.564
Severe Trauma	36(8.4)	13(19.7)	23(6.3)	<0.001
Pulmonary Infection	191(44.4)	56(84.8)	135(37.1)	<0.001
Immune Disease^d^	63(14.7)	22(33.3)	41(11.3)	<0.001
Risk factors (n,%)				
Septic Shock	113(26.3)	34(51.5)	79(21.7)	<0.001
Co-infection with Other Bacteria (Fungi)	166(38.6)	42(63.6)	124(34.1)	<0.001
Receipt of corticosteroids >7 Days	93(21.6)	21(31.8)	72(19.8)	0.029
Chemotherapy	45(10.5)	16(24.2)	29(8)	<0.001
CVC/PICC^e^	175(40.7)	36(54.5)	139(38.2)	0.013
Mechanical Ventilation	159(37)	42(63.6)	117(32.1)	<0.001
Parenteral Nutrition	187(43.5)	41(62.1)	146(40.1)	0.001
Malnutrition	134(31.2)	49(74.2)	85(23.4)	<0.001
ICU Stay > 3 days	102(23.7)	38(57.6)	64(17.6)	<0.001
Renal Dialysis	43(10)	4(6.1)	39(10.7)	0.246
Use of Antifungal Agents	158(36.7)	32(48.5)	126(34.6)	0.032
Surgery	135(31.4)	29(43.9)	106(29.1)	0.017
Various Invasive Catheters	192(44.7)	55(83.3)	137(37.6)	<0.001
Combination of Three or More Antibiotics	107(24.9)	62(93.9)	45(12.4)	<0.001
Use of Carbapenems Before Positive Blood Culture	130(30.2)	51(77.3)	79(21.7)	<0.001
Use of Tigecycline	77(17.9)	27(40.9)	50(13.7)	<0.001
Procalcitonin>100ng/mL	110(25.6)	16(24.2)	94(25.8)	0.786
Outcome (n,%)				
28-day Mortality	75(17.4)	29(43.9)	46(12.6)	<0.001

^a^Includes the following diseases: chronic obstructive pulmonary disease and acute respiratory distress syndrome.

^b^Chronic/Acute renal failure is the permanent or sudden and often temporary loss of kidney function with N waste retention and hypourocrinia.

^c^Including: cholecystitis, pancreatitis and peritonitis.

^d^Includes the following diseases: Including autoimmune diseases(Rheumatoid Arthritis,Systemic Lupus Erythematosus, Multiple Sclerosis)and immunodeficiency diseases(congenital immunodeficiency diseases and AIDS).

^e^CVC, central venous catheter. PICC: Peripherally Inserted Central Catheter.

**Table 2:** Factors associated with 28-day mortality by univariate analysis in patients with KP-BSI, CSKP-BSI and CRKP-BSI.

Variable	All KP-BSI patients 28-days outcome			CSKP-BSI patients 28-days outcome			CRKP-BSI patients 28-days outcome		
	Died (n=75)	Survived (n=355)	P-value	Died (n=46)	Survived (n=318)	P-value	Died (n=29)	Survived (n=37)	P-value
Demographics									
Gender(male:female)	49:26	192:163	0.251	31:15	168:150	0.064	15:14	24:13	0.281
Age (median, range,years)	58(18,95)	59(16, 95)	0.888	59(18, 95)	60(17, 95)	0.981	56(26,95)	55(16,87)	0.344
Length of hospital stay (SD,days)	**20.3(21.9)**	**27.7(31.1)**	**0.049**	18.5(22.9)	25.8(31.2)	0.123	**23.2(20.5)**	**44.3(25.1)**	**0.001**
Underlying comorbidities (n, %)									
Gastrointestinal Perforation	15(20.0)	46(13.0)	0.112	**13(28.3)**	**45(14.2)**	**0.015**	2(6.9)	1(2.7)	0.417
Solid Tumor	8(10.7)	40(11.3)	0.881	6(13)	38(11.9)	0.832	2(6.9)	2(5.4)	0.801
Hematologic Malignancy	**10(13.3)**	**22(6.2)**	**0.032**	2(4.3)	13(4.1)	0.934	8(27.6)	9(24.3)	0.764
Respiratory Dysfunction^a^	**48(64.0)**	**59(16.6)**	**<0.001**	**29(63.0)**	**44(13.8)**	**<0.001**	**19(65.5)**	**15(40.5)**	**0.044**
Hematologic Disease	**28(37.3)**	**58(16.3)**	**<0.001**	9(19.6)	40(12.6)	0.194	19(65.5)	18(48.6)	0.171
Chronic/acute liver disease	31(41.3)	101(28.5)	0.028	13(28.3)	86(27)	0.862	18(62.1)	15(40.5)	0.083
Diabetes Mellitus	27(36.0)	129(36.3)	0.956	21(45.7)	122(38.4)	0.344	6(20.7)	7(18.9)	0.858
Chronic/acute renal failure^b^	**37(49.3)**	**132(37.2)**	**0.050**	23(50)	121(38.1)	0.121	14(48.3)	11(29.7)	0.123
Cardiovascular Disease	**40(53.3)**	**112(31.5)**	**<0.001**	**30(65.2)**	**101(31.8)**	**<0.001**	10(34.5)	11(29.7)	0.681
Gastrointestinal pathology^c^	30(40.0)	102(28.7)	0.055	16(34.8)	89(28)	0.342	14(48.3)	13(35.1)	0.281
Neurological Disease	21(28.0)	89(25.1)	0.597	14(30.4)	81(25.5)	0.474	7(24.1)	8(21.6)	0.809
Severe Trauma	9(12.0)	27(7.6)	0.212	4(8.7)	19(6)	0.478	5(17.2)	8(21.6)	0.657
Pulmonary Infection	**54(72.0)**	**137(38.6)**	**<0.001**	**28(60.9)**	**107(33.6)**	**<0.001**	26(89.7)	30(81.1)	0.335
Immune Disease^d^	15(20.0)	48(13.5)	0.149	5(10.9)	36(11.3)	0.928	10(34.5)	12(32.4)	0.861
Risk factors (n,%)									
Septic Shock	**47(62.7)**	**66(18.6)**	**<0.001**	**31(67.4)**	**48(15.1)**	**<0.001**	16(55.2)	18(48.6)	0.599
Co-infection with Other Bacteria (Fungi)	**45(60.0)**	**121(34.1)**	**<0.001**	**23(50)**	**101(31.8)**	**0.015**	22(75.9)	20(54.1)	0.068
Receipt of corticosteroids >7 Days	**28(37.3)**	**65(18.3)**	**<0.001**	**15(32.6)**	**57(17.9)**	**0.019**	**13(44.8)**	**8(21.6)**	**0.045**
Chemotherapy	10(13.3)	35(9.9)	0.372	3(6.5)	26(8.2)	0.699	7(24.1)	9(24.3)	0.986
CVC/PICCe	33(44.0)	142(40.0)	0.522	16(34.8)	123(38.7)	0.611	17(58.6)	19(51.4)	0.556
Mechanical Ventilation	**49(65.3)**	**110(31.0)**	**<0.001**	**27(58.7)**	**90(28.3)**	**<0.001**	22(75.9)	20(54.1)	0.068
Parenteral Nutrition	**44(58.7)**	**143(40.3)**	**0.004**	**27(58.7)**	**119(37.4)**	**0.006**	17(58.6)	24(64.9)	0.604
Malnutrition	**32(42.7)**	**102(28.7)**	**0.018**	11(23.9)	74(23.3)	0.923	21(72.4)	28(75.7)	0.764
ICU Stay > 3 days	**34(45.3)**	**68(19.2)**	**<0.001**	**16(34.8)**	**48(15.1)**	**0.001**	18(62.1)	20(54.1)	0.513
Renal Dialysis	9(12.0)	34(9.6)	0.525	6(13)	33(10.4)	0.585	3(10.3)	1(2.7)	0.197
Use of Antifungal Agents	33(44.0)	125(35.2)	0.151	19(41.3)	107(33.6)	0.308	14(48.3)	18(48.6)	0.976
Surgery	18(24.0)	117(33)	0.129	**5(10.9)**	**101(31.8)**	**0.004**	13(44.8)	16(43.2)	0.898
Various Invasive Catheters	41(54.7)	151(42.5)	0.055	14(30.4)	123(38.7)	0.281	27(93.1)	28(75.7)	0.059
Combination of Three or More Antibiotics	**33(44.0)**	**74(20.8)**	**<0.001**	6(13)	39(12.3)	0.881	27(93.1)	35(94.6)	0.801
Use of Carbapenems Before Positive Blood Culture	**41(54.7)**	**89(25.1)**	**<0.001**	15(32.6)	64(20.1)	0.055	**26(89.7)**	**25(67.6)**	**0.034**
Use of Tigecycline	18(24.0)	59(16.6)	0.130	**2(4.3)**	**48(15.1)**	**0.048**	**16(55.2)**	**11(29.7)**	**0.037**
Procalcitonin>100ng/mL	23(30.7)	87(24.5)	0.267	14(30.4)	80(25.2)	0.445	9(31.0)	7(18.9)	0.254
CRKP vs. CSKP	**29(38.7)**	**37(10.4)**	**<0.001**	–	–	–	–	–	–

^a^Includes the following diseases: chronic obstructive pulmonary disease and acute respiratory distress syndrome.

^b^Chronic/Acute renal failure is the permanent or sudden and often temporary loss of kidney function with N waste retention and hypourocrinia.

^c^Including: cholecystitis, pancreatitis and peritonitis.

^d^Includes the following diseases: Including autoimmune diseases(Rheumatoid Arthritis,Systemic Lupus Erythematosus, Multiple Sclerosis)and immunodeficiency diseases(congenital immunodeficiency diseases and AIDS).

^e^CVC, central venous catheter; PICC, Peripherally Inserted Central Catheter.

Bold content indicates that the variable has statistical significance when compared between the two groups of data.

### Risk factors for 28-day mortality in KP-BSI patients

**Figure 4:**
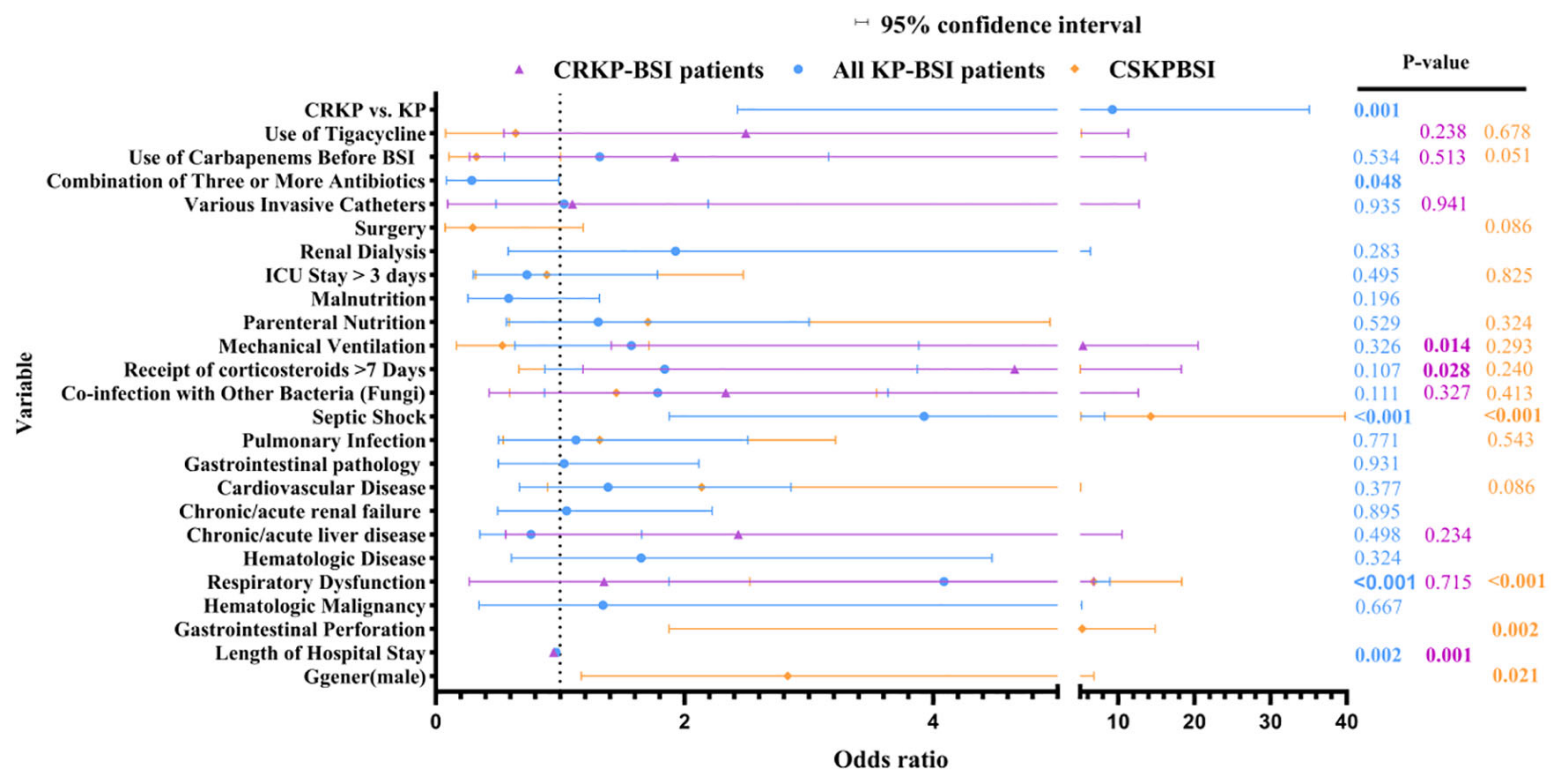
Factors associated with 28-day mortality by multivariate analysis. Length of Hospital Stay (All KPBSI)(95% CI: 0.968–0.988), Length of Hospital Stay(CRKPBSI)(95% CI: 0.947–0.979). Total cohort: 75 death events, with 21 covariates included in the final model, EPV = 3.6; CRKP-BSI subgroup: 29 death events, 9 covariates, EPV = 3.2; CSKP-BSI subgroup: 46 death events, 14 covariates, EPV = 3.3. Although the EPV of the CRKP-BSI subgroup is below the traditional threshold of 10, we performed a sensitivity analysis using Firth penalized likelihood regression, and the direction and significance of the effects did not change substantially, with robust results.

We compared deceased and surviving patients among all KP-BSI patients within 28 days post-infection. The prevalence of underlying diseases was significantly higher in deceased patients than in survivors. Specifically, the underlying diseases were identified as risk factors for mortality in all KP-BSI infections (*P ≤* 0.05): hematologic malignancy (13.3% vs. 6.2%), respiratory dysfunction (64.0% vs. 16.6%), hematologic disease (37.3% vs. 16.3%), chronic/acute liver disease (41.3% vs. 28.5%), chronic/acute renal failure (49.3% vs. 37.2%), cardiovascular disease (53.3% vs. 31.5%), and pulmonary infection (72.0% vs. 38.6%). Moreover, the proportion of risk factors, including septic shock, co-infection with other bacteria (or fungi), receipt of corticosteroids for more than 7 days, mechanical ventilation, parenteral nutrition, malnutrition, ICU stay exceeding 3 days, combination of three or more antibiotics, use of carbapenems before positive blood culture, and CRKP vs. KP infection, was also higher in deceased patients than in survivors (*P ≤* 0.05). The detailed results are presented in **Table 2**. Multivariate logistic regression analysis identified respiratory dysfunction (OR, 4.09; 95%CI, 1.88–8.90; *P* < 0.001), septic shock (OR, 3.93; 95% CI, 1.88–8.21; *P* < 0.001), and CRKP vs. KP BSI infections (OR, 9.23; 95% CI, 2.43–35.12; *P* = 0.001) as independent risk factors for mortality in all KP-BSI infections. Conversely, increased length of hospital stay (OR, 0.968; 95% CI, 0.949–0.988; *P* = 0.002) and the combination of three or more antibiotics (OR, 0.29; 95% CI, 0.09–0.99; *P* = 0.048) emerged as protective factors against mortality in all KP-BSI infections (**Figure 4**).

We further compared deceased and surviving patients among CRKP-BSI and CSKP-BSI patients within 28 days post-infection. Although the prevalence of underlying diseases was higher in deceased patients than in survivors, only respiratory dysfunction (65.5% vs. 40.5%) was identified as a significant risk factor for mortality in CRKP-BSI infections (*P* = 0.044), however, gastrointestinal perforation (28.3% vs. 14.2%), respiratory dysfunction(63.0% vs. 13.8%), cardiovascular disease(65.2% vs. 31.8%) and pulmonary infection(60.9% vs. 33.6%) were identified as a significant risk factor for mortality in CSKP-BSI infections(*P ≤* 0.05). In terms of risk factors, receipt of corticosteroids > 7 days, use of carbapenems before positive blood culture and use of tigecycline were identified as significant risk factors for mortality in CRKP-BSI infections (*P* < 0.05); septic shock, co-infection with other bacteria (fungi), receipt of corticosteroids >7 days, mechanical ventilation, parenteral nutrition, ICU stay > 3 days were identified as significant risk factors for mortality in CSKP-BSI infections (*P* < 0.05). The detailed results are presented in **Table 2**. Multivariate logistic regression analysis identified mechanical ventilation (OR, 5.38; 95% CI, 1.41–20.50; *P* = 0.014) and receipt of corticosteroids > 7 days (OR, 4.66; 95% CI, 1.18–18.29; *P* = 0.028) as independent risk factors for mortality in CRKP-BSI infections. Conversely, an increased length of hospital stay (OR, 0.947; 95% CI, 0.915–0.979; *P* = 0.001) was found to be a protective factor against mortality in CRKP-BSI infections (**Figure 4**). Septic shock (OR, 14.27; 95% CI, 5.12–39.78; *P* < 0.001) and respiratory dysfunction (OR, 6.81; 95% CI, 2.53–18.37; *P* < 0.001), gastrointestinal perforation (OR, 5.28; 95% CI, 1.88–14.86; *P* = 0.002), and gender (male vs. female) (OR, 2.83; 95% CI, 1.17–6.84; *P* = 0.021) were identified as independent risk factors for mortality in CSKP-BSI infections (**Figure 4**).

### Survival analysis

**Figure 5:**
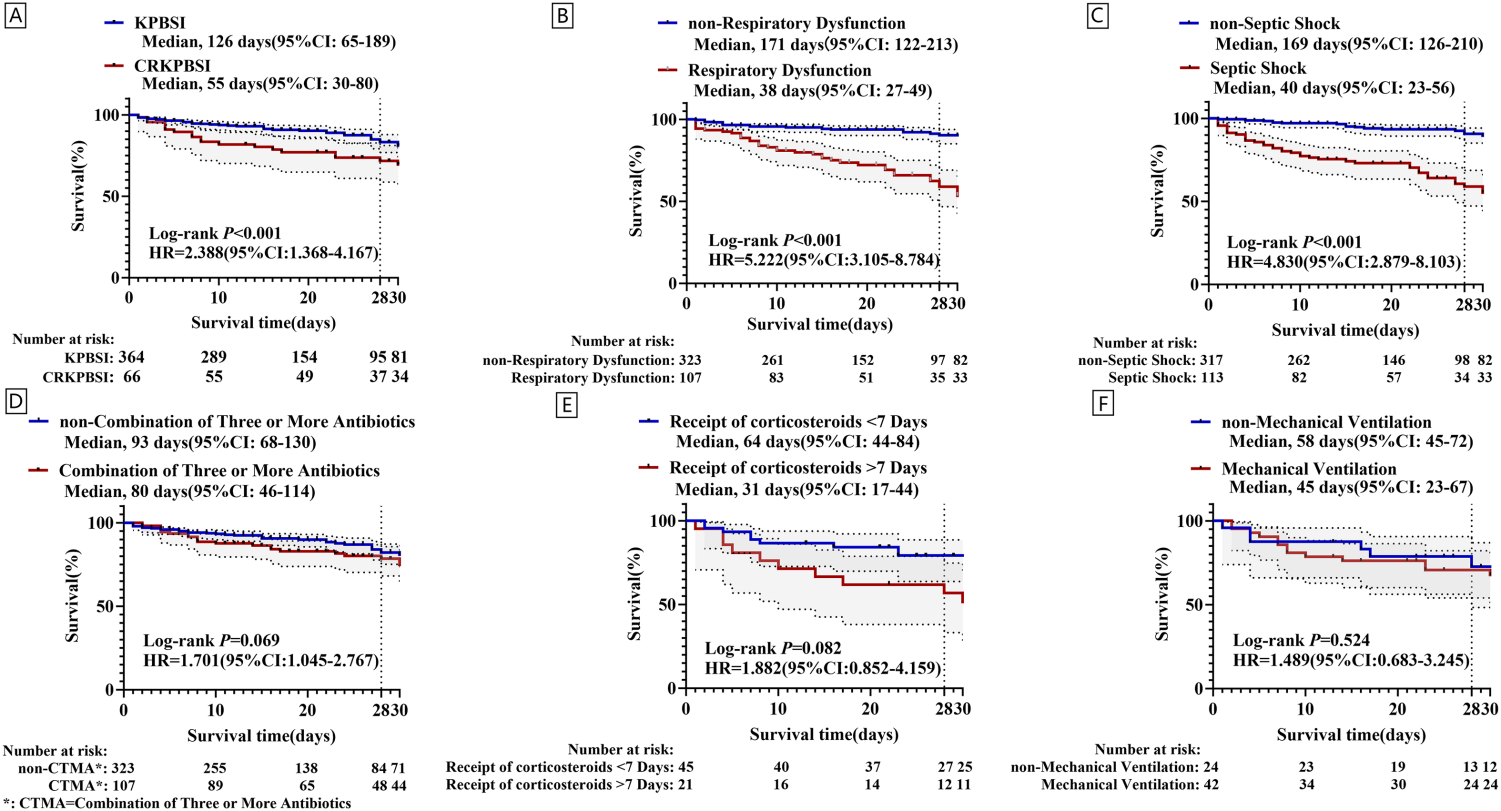
Kaplan-Meier survival analysis was used to evaluate the impact of independent risk factors on 28-day mortality and survival duration among patients with sepsis.

Kaplan-Meier survival analysis was utilized to assess the influence of independent risk factors on 28-day mortality and survival duration among sepsis patients. Mortality was designated as the endpoint, and the Log-Rank test was employed for data analysis. The survival curve analysis indicated that CRKP versus KP bloodstream infections, respiratory dysfunction, and septic shock were associated with reduced survival times in patients with KPBSI infections (**Figures 5A–C**). In contrast, mechanical ventilation, corticosteroid use for more than 7 days, and combination therapy with three or more antibiotics had no significant effect on survival time (**Figures 5D–F**).

### Results of molecular typing, resistance genes, and virulence-associated genes

**Figure 6:**
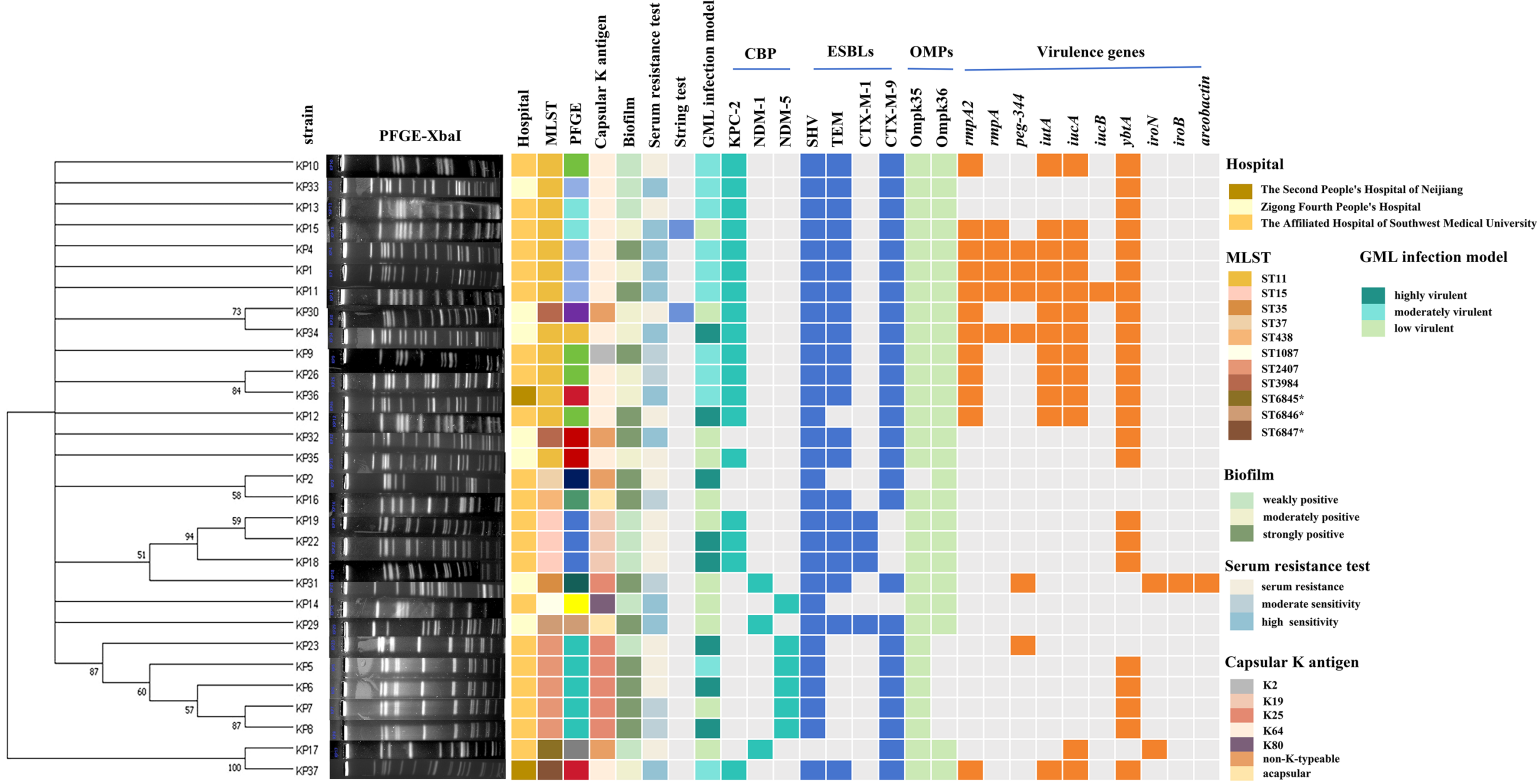
Phylogenetic tree of 30 CRKP strains constructed using MEGA software based on MLST data. Strain numbers, PFGE lane, Hospital: hospital information, MLST, multilocus sequence typing; PFGE, PFGE typing; CBP, carbapenemase; ESBLs, extended spectrum beta-lactamases; OMPs, outer membrane proteins; Virulence genes; GML, Galleria mellonella Larval Infection Model.

In this study, a total of 30 CRKP strains were chosen from three hospitals for molecular epidemiological analysis, including 21 strains from the Affiliated Hospital of Southwest Medical University, 7 strains from Zigong Fourth People’s Hospital, and 2 strains from Neijiang Second People’s Hospital. A total of 14 distinct subtypes were identified through PFGE. MLST revealed eight sequence types (STs), with ST11 being the most prevalent, accounting for 43.33% (13/30) of the strains. Additionally, three novel STs were identified: ST6845(KP17), ST6846(KP29), and ST6847(KP37). The detection rates of carbapenemase-resistant genes were: *blaKPC-2*(60.0%,18/30), *blaNDM-5*(20.0%, 6/30), and *blaNDM-1*(10.0%, 3/30). For the extended-spectrum β-lactamase (ESBL) resistance genes, the detection rates were *blaSHV* (96.7%, 29/30), *blaCTX-M* (73.3%, 22/30), *blaTEM* (70.0%, 21/30), and *blaCTX-M-1* (13.3%, 4/30). The outer membrane porin genes Ompk35 and Ompk36 were detected in 96.7% (29/30) and 83.3% (25/30) of the strains, respectively. Among the 30 CRKP strains causing bloodstream infections, 2 strains lacked the capsular gene of *wzi*, while 28 strains carrying the *wzi* gene were classified into five distinct serotypes, the capsular serotype K64 had the highest proportion at 46.67% (14/30), as shown in **Figure 6**. Furthermore, nine common virulence genes were detected among the 30 CRKP strains causing bloodstream infections, with the following detection rates: *rmpA2*(40.0%,12/30), *rmpA*(20.0%, 6/30), *peg-344*(26.7%, 8/30), *iutA*(40.0%, 12/30); *iucA*(43.3%,13/30), *YbtA*(76.7%, 23/30), *iroN*(6.7%, 2/30), *iroB*(3.3%,1/30), and *aerobactin*(3.3%, 1/30). The detailed results are presented in **Figures 6**.

### Phenotypic characteristics related to antimicrobial resistance and virulence

The biological characteristics of 30 bacterial strains were comprehensively analyzed. The modified carbapenem inactivation method(mCIM) in conjunction with the EDTA disk synergy test(eCIM) was utilized to perform a preliminary screening for carbapenemase production among 30 CRKP isolates, the results indicated that 27 isolates(90.0%) were positive for mCIM, while 8 isolates(29.6%) were positive for eCIM. Regarding biofilm formation, 25 of the 30 strains demonstrated the ability to form biofilms, resulting in a positivity rate of 83.3%. Specifically, the distribution of biofilm formation intensity was as follows: 36.7% (11/30) of the strains exhibited strong positive, 40.0% (12/30) showed moderate positive, and 6.67% (2/30) displayed weak positive. In the string test, only 2 strains tested positive, characterized by mucoid filament lengths exceeding 5 mm, indicative of a hypermucoviscous phenotype, with a positivity rate of 6.67% (2/30). In the Galleria mellonella larval infection model, an inoculum of 1 × 10^5^ CFU showed that eight test isolates were as virulent as the highly virulent reference strain NTUH-K2044; eleven isolates exhibited virulence intermediate between NTUH-K2044 and the low-virulence reference strain ATCC 700603; and the remaining eleven isolates were statistically indistinguishable from ATCC 700603 in virulence (**Supplementary Figure 1**). In the serum resistance assays, 11 strains(36.7%) exhibited serum resistance (grades 5 and 6), 6 strains(20.0%) exhibited serum moderate sensitivity (grades 3 and 4), and 13 strains(43.3%) exhibited serum high sensitivity (grades 1 and 2) (**Supplementary Figure 2**). The other detailed results are presented in **Figure 6**.

## Discussion

This study reveals that the hepatobiliary surgery and hematology wards are high-incidence areas for KP and CRKP bloodstream infections. In light of these observations, it is of utmost importance to strengthen targeted infection control measures in these high-risk wards in order to effectively reduce the incidence of CRKP and KP-BSI, thereby ensuring patient safety in the southern region of Sichuan Province, China. Additionally, the ICU has emerged as the ward with the highest incidence of CRKP-BSI in the southern region of Sichuan Province. This finding is in broad alignment with the results of other relevant studies conducted both domestically and internationally (Zhong et al., 2025; De Pascale et al., 2025; Cao et al., 2025). It underscores the necessity for the ICU to remain a focal point for the prevention and control of CRKP-BSI, with a continued emphasis on the reinforcement of targeted infection control measures.

The results of this study demonstrate that the overall mortality rate among patients with KP-BSI was higher than that reported by Roach DJ, et al. (14.4%) (Roach et al., 2024) and Fostervold A, et al(12.5%) (Fostervold et al., 2024) but lower than that reported by Yu et al. (30.0%) (Yu et al., 2024) and Wu X, et al(30.4%) (Wu et al., 2021). Although CRKP accounted for only 15.3% (66/430) of bloodstream isolates, a proportion similar to that reported in Southwest China (Long et al., 2025) and Turkey (Kilinc, 2025), it was significantly lower than the incidence rates in Northern China (Li et al., 2020), Western Greece (Lagadinou et al., 2024), and India (Jain et al., 2025). However, the 28-day mortality rate for CRKP was as high as 43.9%, consistent with the results of Cheng et al. (46.0%) (Cheng et al., 2024), higher than that of Wang et al. (34.0%) (Wang et al., 2022), and lower than that of Xu et al. (54.3%) (Xu et al., 2017). The observed variations in mortality rates across these studies may be attributed to differences in the clinical profiles of the patient populations, regional disparities, and the distinct methodologies employed in each investigation. Given these considerations, conducting multicenter studies across diverse geographical regions is essential for enhancing the precision of prevention and control measures targeting KP-BSI.

The carbapenem-resistance trajectory of bloodstream Klebsiella pneumoniae isolates documented in southern Sichuan parallels that observed across south-western China (Long et al., 2025), yet remains markedly lower than the rates reported for Chengdu, China (Deng et al., 2022); this divergence is presumably attributable to the distinct patient case-mix encountered by Chengdu’s tertiary referral centers. Among carbapenem-resistant Klebsiella pneumoniae (CRKP), the rates of resistance to β-lactams and quinolones observed in this study align with those reported from Zhejiang (Zhong et al., 2025) and Taiwan (Hsu et al., 2021); However, the resistance rate to fluoroquinolones was significantly lower than that reported in the Hebei region of China (Cheng et al., 2024). The aminoglycoside resistance rate was comparable to that in Hebei, lower than in Zhejiang, and higher than in Taiwan, while the tigecycline resistance rate was lower than all the aforementioned regions. Overall, these data underscore that even within CRKP populations, the accompanying resistance profiles vary markedly across different geographical areas in China. Therefore, multicenter studies must incorporate regional characteristics to accurately inform targeted interventions aimed at curbing the spread of resistant strains.

Analysis of clinical characteristics from KP-BSI patients in this study revealed that the incidence of underlying diseases and risk factors was significantly higher in CRKP-BSI patients than in CSKP-BSI patients. Pulmonary infection, ICU stay > 3 days, various invasive catheters, and combination of three or more antibiotics were identified as independent risk factors for CRKP-BSI infection. These independent risk factors are largely consistent with findings from multiple domestic (Cheng et al., 2024; Chang et al., 2020; Chen et al., 2022; Li et al., 2020; Liu et al., 2022; Xiao et al., 2020; Zhu et al., 2023; Liang et al., 2022; Cienfuegos-Gallet et al., 2022) and international studies (Şimşek Bozok et al., 2025; Gupta et al., 2021), which may be attributed to the ICU being a high-risk environment for multidrug-resistant bacteria, where invasive procedures increase infection susceptibility and combination antibiotic use exerts selective pressure. Multivariate analysis indicated that mechanical ventilation and receipt of corticosteroids >7 days were independent risk factors for mortality in CRKP-BSI patients, whereas septic shock, respiratory dysfunction, gastrointestinal perforation, and gender(male) were independent risk factors for mortality in CSKP-BSI patients. These risk factors are largely consistent with those reported in the majority of previous domestic (Chang et al., 2020; Chen et al., 2022; Zhang et al., 2020) and international literature (Şimşek Bozok et al., 2025). Nevertheless, discrepancies do exist (Meng et al., 2023; Onorato et al., 2022; Ying et al., 2022; Lumbreras-Iglesias et al., 2023; Aslan et al., 2022), which can largely be explained by heterogeneity among the study cohorts—specifically, differences in the spectrum of underlying comorbidities and the geographic locations from which patients were recruited. Additionally, the apparent “protective” effect of length of hospital stay mainly reflects the fact that such patients have typically entered a clinically stable phase and are ready for ICU transfer, so their early mortality risk has already declined substantially, giving rise to immortal-time bias. Likewise, the combination of three or more antibiotics usually represents early empirical broad-spectrum therapy initiated when patients are still in the initial stage of infection with relatively preserved organ function; subsequent de-escalation to targeted treatment can then improve outcome, introducing time-dependent confounding. These phenomena exemplify the common “time-zero” misalignment in observational studies. To eliminate such biases, we recommend that future investigations employ prospective designs for validation. This study also employed Kaplan–Meier survival analysis to assess the impact of independent risk factors on 28-day mortality and survival time in septic patients. In this study focusing on patients with CRKP-BSI, no independent risk factors significantly associated with patient survival time were identified. This result may be attributed to the heavy burden of underlying diseases and generally poor prognosis among the study population, as well as the relatively limited sample size, which reduced statistical power and thus may have hindered the detection of potential influencing factors. In the future, our research team will further expand this line of investigation by extending multicenter collaboration and prolonging the observation period to increase the sample size, thereby enhancing the statistical power and reliability of the study conclusions. **Table 3** compares the independent risk and mortality factors established in this study with those reported in the literature. In summary, the risk factor profiles for mortality differ between KP-BSI with distinct resistance phenotypes. It is clinically essential to establish individualized diagnosis, treatment, and prognostic evaluation systems tailored specifically for CRKP and CSKP infections, respectively, in order to optimize the timing of interventions and improve patient outcomes.

**Table 3:** Protective factor and predictors of 28 or 30-days mortality in others studies.

Authors	Country or region	Study period	Study design	Samples	No of samples	Mortality (28 or 30 days))	Independent risk factors for CRKP BSI	Protective factors of 28-day mortality	Predictors of 28-day mortality	Reference
Cheng et al	China	2016-2020	Retrospective, single-center study	Adults, CRKP(50) and CSKP(84)	134	46.0%	gastric catheterization, prior ICU hospitalization, and detection of CRKP in non-blood sites		microbiologic eradication after 6 days, high Pitt bacteremia score, and inappropriate empirical treatment after BSIs	Cheng et al., 2024
Şimşek Bozok et al	Türkiye	2021-2023	Retrospective, single-center study	Adults, CRKP(66) and CSKP(41)	107	80.3%	acute renal failure, mechanical ventilation, post-earthquake period and use of carbapenem and other beta-lactam antibiotics		intensive care unit follow-up, intubation, MV monitoring and concomitant pneumonia	Şimşek Bozok et al., 2025
Yu et al	China	2015-2022	Retrospective, single-center study	Infants, CRKP(96)	96	27.1%			Concurrent meningitis, concurrent necrotizing enterocolitis (NEC) and the maximum vasoactive-inotropic score (VIS) value within 48 h of onset	Yu et al., 2024
Chang et al	China	2014-2018	Retrospective cohort study, single-center study	CRKP(46) and CSKP(239) (adult patients)	285	50%	ICU stay, exposure to antifungals, exposure to quinolones, and the number of isolated bacterial species from the patient ≥ 3		hematological tumor, chronic lung disease, and septic shock	Chang et al., 2020
Li et al	China	2014-2019	Retrospective, observational, single-center study	CRKP(164) and CSKP(328) (adult patients)	492	43.9%	KP detection in other sites, blood purification, bronchoscopy, surgery, carbapenem use, tigecycline use		Previous hospitalization, long hospitalization, bone marrow puncture, use of β-lactamase inhibitor (P = 0.005, OR 3.890)	Li et al., 2020
Chen et al	China	2012-2019	Retrospective, single-center study	CRKP(212) and CSKP(496) (adult patients)	706	42.5%	Hematologic malignancies and ICU acquired infection	Combination with high doses of carbapenem	Corticosteroids use preceding infection onset, Inadequate empirical antibiotic therapy, Severe sepsis/septic shock	Chen et al., 2022
Xiao et al	China	2013-2015	Retrospective, single-center study	CRKP(104) and CSKP(203) (adult patients)	307	55.8%	previous gastric catheterization, previous use of carbapenems, hypoproteinemia, and high Acute Physiologic Assessment and Chronic Health Evaluation II scores		severe illness, and tigecycline therapy	Xiao et al., 2020
Zhu et al	China	2020	Retrospective, observational, single-centered study	CRKP(50) and CSKP(58)(>18 years patients)	108	36.0%	prior ICU hospitalization and use of carbapenems			Zhu et al., 2023
Liu et al	China	2018-2020	Retrospective, single-center study	CRKP(77) and CSKP(433)(>18 years patients)	510	44.6%	a long hospital stay before BSI, ICU hospitalization history, and prior use of carbapenems and antifungals			Liu et al., 2022
Liang et al	China	2010-2018	Retrospective, multicenter study (10 hospitals)	CRKP(56) and CSKP(47)(all patients)	103	35.7%	tracheal cannula or tracheotomy (within 30 days), active empiric antibiotic therapy, and tigecycline treatment		tracheal cannula or tracheotomy (within 30 days), severe sepsis, and length of hospitalization after the onset of BSI	Liang et al., 2022
Gupta et al	India	2014-2015	Retrospective, single-center study	CRKP(85) and CSKP(26)(all patients)	111	42.4%	prior carbapenem use, presence of Foley catheter and admission to gastroenterology service			Gupta et al., 2021
Meng et al	China	2018-2021	Retrospective, single-center study	CRKP(70)(all patients)	70	39.4% (neonates), 43.2% (older children)		a higher platelet count, the use of carbapenems , and appropriate targeted antimicrobial treatment (non-neonate)	a higher Pitt bacteremia score (neonate)	Meng et al., 2023
Lumbreras-Iglesias et al	Spain	2014-2019	Retrospective, single-center study	CRKP(76)(all patients)	76	31.6%			Hematological malignancy, Lower respiratory system infection, Inadequate therapy, ST147 Clone	Lumbreras-Iglesias et al., 2023
Cienfuegos-Gallet et al	China	2011-2020	retrospective cohort study, single-center study	CRKP(29) and CSKP(223)(≥ 65 years)	252	48.3%	Hypertension, exposure to carbapenems, and ICU stay		isolation of CRKP and KP isolated in ICU	Cienfuegos-Gallet et al., 2022
Yang et al	China	2017-2020	Retrospective, single-center study	CRKP(146) and CSKP(39)(all patients)	185	20.5%			Age, Urinary catheter	Yang et al., 2022
Zhang et al	China	2013-2019	Retrospective, single-centered study	CRKP(108) and CSKP(388)(all patients)	496	38.0%			higher Charlson Comorbidity Index score, respiratory failure, renal failure, septic shock, mechanical ventilation and CRKP infection	Zhang et al., 2020
Ying et al	China	2016-2020	Retrospective, single-center study	CSKP(72)(>16years patients)	72	37.5%			SOFA score, Age, Total adipose tissue	Ying et al., 2022
Onorato et al	Italy	2020	Retrospective, multicenter study	CSKP(154)(all patients)	154	32.5% (7 days), 41.9% (90 days)			the presence of at least one risk factor	Onorato et al., 2022
Aslan et al	Turkey	2014-2018	Retrospective, observational, single-center study	CSKP(124)(>18 years patients)	124	39.7%			INCREMENT CPE mortality score, sepsis at BSI onset and inappropriate therapy	Aslan et al., 2022
This study	China	2020-2024	Retrospective, observational, multicenter, cohort study	CRKP(66) and CSKP(364), >16 years patients	430	KP-BSI(17.4%),CRKP(43.9%) and CSKP(12.6%)	Pulmonary infection, ICU stay > 3 days, various invasive catheters, and combination of three or more antibiotics	Prolonged length of hospital stay(days)	mechanical ventilation and receipt of corticosteroids >7 days	This study

CRKP, carbapenem-resistant K. pneumoniae; BSI, bloodstream infections; CSKP, carbapenem-susceptible Klebsiella pneumoniae; APACHE, Acute Physiology and Chronic Health Evaluation; ICU, Intensive care unit; SOFA, Sequential Organ Failure Assessment.

This study analyzed the molecular characteristics of CRKP in the Southern Sichuan Province of China. The results revealed that ST11 was the predominant clone in this region, a finding consistent with most domestic reports (Hu et al., 2020; Wu et al., 2024; Cienfuegos-Gallet et al., 2022; Yang et al., 2022; Wang et al., 2023) but differing from reports in Europe (Budia-Silva et al., 2024). Notably, three novel sequence types (STs) were identified for the first time: ST6845 (KP17), ST6846 (KP29), and ST6847 (KP37). The emergence of these new STs suggests that the local CRKP population may possess high genetic diversity and evolutionary dynamics, highlighting the necessity for sustained local molecular epidemiological surveillance. Regarding carbapenemase resistance genes, *blaKPC-2* was the predominant genotype, aligning with the overall national trend (Wu et al., 2024; Shi et al., 2024). However, a distinctive local feature was the higher detection rate of *blaNDM-5* compared to *blaNDM-1*, a pattern that differs from some regional reports (Cienfuegos-Gallet et al., 2022) while being consistent with others (Yuan et al., 2024). Furthermore, co-carriage of carbapenemase genes and extended-spectrum β-lactamase (ESBL) genes was observed in all isolates (100%). Concurrently, the rates of mutation or loss of the outer membrane porin genes *ompK35* and *ompK36* were 96.7% (29/30) and 83.3% (25/30), respectively. The co-existence of these multiple resistance mechanisms strongly implies that strains with combined resistance mutations have been selectively enriched under antimicrobial selective pressure, which is likely a key factor contributing to the high resistance rates of CRKP in this region.

Previous studies have indicated that the ST11 lineage of KP is often associated with a hypervirulent phenotype (Zhao et al., 2019; Shao et al., 2021). Based on this, we systematically evaluated virulence-associated characteristics of CRKP isolates, including their biofilm formation capacity, serum resistance, the string test, as well as their capsular and virulence gene profiles. Capsular serotyping revealed that K64 was the predominant type among the locally prevalent strains. This finding is consistent with some reports (Zhou et al., 2021; Hu et al., 2024) but differs from others (Shao et al., 2021; Li et al., 2019). Analysis of virulence genes further uncovered a distinct local genetic profile: the most prevalent virulence gene was *ybtA* (76.67%, 23/30), followed by *iucA* (43.33%, 13/30), *iutA* (40%, 12/30), and *rmpA2* (40%, 12/30). This distribution pattern differs significantly from those reported in other studies (Jin et al., 2022). Phenotypic analysis indicated that the CRKP isolates in this study exhibited a strong capacity for biofilm formation (positivity rate of 83.33%), which may be related to the fact that these bacteria were isolated from bloodstream infections. As reported in previous studies, strains isolated from blood are more capable of forming biofilms (Ashwath et al., 2022). Serum resistance assays showed that 36.67% (11/30) of the strains exhibited high serum resistance, while the positivity rate in the string test was considerably lower, at only 6.68% (2/30). This is consistent with another study reported in the southwestern region of China (Wang et al., 2023). The *Galleria mellonella* infection model confirmed the *in vivo* virulence of the strains, with 63.33% (19/30) identified as hypervirulent or moderately virulent. Notably, among these hypervirulent/moderately virulent strains, ST11 (accounting for 57.89%) and K64 (accounting for 63.16%) were the predominant types. Furthermore, 57.89% of these strains co-carried a combination of genes regulating the hypermucoviscous phenotype (rmpA/rmpA2) and genes associated with siderophore production (iutA/iucA/peg-344), however, all strains carrying this gene set were, without exception, classified as at least moderately virulent. Conversely, strains lacking the complete combination could still display moderate-to-high virulence; therefore, the absence of these genes cannot be used to exclude the hypervirulent potential. In summary, we emphasize that the definitive classification of hypervirulent K. pneumoniae (hvKP) requires integration of *in vivo* infection model data, while isolates simultaneously possessing rmpA/rmpA2 together with iutA/iucA/peg-344 can be preliminarily regarded as moderately to highly virulent. In conclusion, the accurate identification of hvKP should not rely solely on molecular typing (e.g., ST and capsular serotype), the hypermucoviscous phenotype, or the presence of individual virulence genes. Instead, it necessitates a comprehensive assessment incorporating results from animal model experiments. Concurrently, our findings provide molecular evidence confirming significant regional heterogeneity in the molecular epidemiology of CRKP, despite the shared phenotype of carbapenem resistance. This underscores the critical importance of conducting multicenter molecular epidemiological studies tailored to specific regions for the effective containment of local resistant strain dissemination.

However, this study also has several limitations. First, its retrospective design relies on the completeness and accuracy of electronic medical records, which may introduce information bias. Second, despite being a five-year multicenter study, the number of CRKP isolates ultimately included for in-depth molecular and phenotypic analysis remains relatively limited, which may affect the statistical power and generalizability of the findings to some extent. Third, potential heterogeneity in infection diagnosis, clinical practices, and prevention-control strategies among the three participating hospitals may also pose challenges to the consistent interpretation of the results. Finally, by restricting inclusion to patients aged 16 years and older, the conclusions of this study primarily reflect the epidemiological and resistance characteristics of CRKP bloodstream infections in the adult population and may not be directly extrapolated to pediatric and adolescent populations. To address these limitations, future studies could expand the multicenter collaborative network, extend the study duration to include a larger sample of isolates, and consider prospective designs. Additionally, conducting research specifically focused on pediatric populations would help to achieve a more comprehensive understanding of the epidemiology of CRKP infections.

## Conclusions

The ICU and hematology wards exhibited the highest isolation rates of CRKP. The 28-day mortality rate among patients with CRKP-BSI reached 43.9%. Multivariate analysis identified pulmonary infection, ICU stay >3 days, various invasive catheters, and combination of three or more antibiotics as independent risk factors for CRKP-BSI. Mechanical ventilation and receipt of corticosteroids >7 days emerged as independent predictors of death and can serve as high-risk signals for mortality in patients with CRKP-BSI. We recommend early intensified infection control and corticosteroid tapering strategies. Molecular epidemiology revealed ST11 as the dominant clone, with three novel sequence types identified: ST6845, ST6846, and ST6847. Carbapenemase genes were primarily *blaKPC-2* and *blaNDM-5*, while extended-spectrum β-lactamase genes were mainly *blaSHV* and *blaCTX-M*. The predominant capsular serotype was K64, and *ybtA* had the highest detection rate among virulence genes. Phenotypically, 83.3% of strains formed biofilms, only 2 isolates exhibited the hypermucoviscous phenotype (string-test positive), and 36.7% displayed serum resistance. Based on the above information, we suggest that high-risk wards need tailored empirical treatment protocols, such as early intensified infection control and corticosteroid tapering strategies, and the necessity for ongoing molecular surveillance of emerging clones. This study comprehensively delineates the clinical and molecular epidemiology of CRKP-BSI in Southern Sichuan province of China, providing robust scientific evidence for clinical management and infection-control strategies against CRKP bloodstream infections in this region.

## Data Availability

The datasets presented in this study can be found in online repositories. The names of the repository/repositories and accession number(s) can be found in the article/**Supplementary Material**. Further inquiries can be directed to the corresponding author. The sequences datasets can be found in the NCBI repository, Accession to SRA data: (https://www.ncbi.nlm.nih.gov/bioproject/). BioProject: PRJNA1266376 and PRJNA1266379.
