# Supplementary material for: Clinical and molecular characteristics, antibiotic susceptibility, risk factors and predictors of mortality in carbapenem-resistant klebsiella pneumoniae bloodstream infections in Southern Sichuan, China: a 5-year multicenter study
Source: Front Cell Infect Microbiol. 2026 Feb 23;16:1741170. doi: 10.3389/fcimb.2026.1741170 (PMC12968308; doi:10.3389/fcimb.2026.1741170)
Supplement: Supplementary file 1 [file Table1.docx]

Supplementary Material

# Supplementary Figures and Tables

**1.1 Supplementary Figures**

**Supplementary Figure 1.** Virulence assessment of NTUH-K2044 and 30 CRKP BSI isolates using the Galleria mellonella larval infection model.

Footnote：NTUH-K2044：Klebsiella pneumoniae, the highly virulent reference strain ；ATCC 700603：Klebsiella pneumoniae, the low-virulence reference strain ；PBS：phosphate-buffered saline，and served as the blank control.

**Supplementary Figure 2.** Serum resistance testing of 30 CRKP BSI strains in comparison with NTUH-K2044

Footnote：NTUH-K2044：Klebsiella pneumoniae, the highly virulent reference strain ；ATCC 700603：Klebsiella pneumoniae, the low-virulence reference strain ；PBS：phosphate-buffered saline，and served as the blank control.

**1.2 Supplementary Tables.**

**Supplementary Table 1.** Primers used in this study.

| Gene | Primer sequence (5’-3’) | Fragment length（bp） | Annealing temperature（℃） | Purpose |
| --- | --- | --- | --- | --- |
| **Resistance gene** | |  |  |  |
| KPC | F: CGTCTAGTTCTGCTGTCTTG | 798 | 60 | PCR |
|  | R: CTTGTCATCCTTGTTAGGCG |  |  |  |
| NDM | F: GGTTTGGCGATCTGGTTTTC | 621 | 58 | PCR |
|  | R: CGGAATGGCTCATCACGATC |  |  |  |
| IMP | F: GGAATAGAGTGGCTTAACTCTC | 232 | 54 | PCR |
|  | R: GGTTTAACAAAACAACCACC |  |  |  |
| VIM | F:TGGTGATGAGTTGCTTTTGA | 306 | 57 | PCR |
|  | R: GCAGCACCAGGATAGAAGAG |  |  |  |
| OXA-48 | F:GCGTGGTTAAGGATGAACAC | 438 | 56 | PCR |
|  | R: CATCAAGTTCAACCCAACCG |  |  |  |
| OmpK35 | F:AACTTATTGACGGCAGTGGC | 1077 | 66 | PCR |
|  | R:TTGGTAAACGATACCCACGG |  |  |  |
| OmpK36 | F:GCAGTGGCATAATAAAAGGCA | 1119 | 62 | PCR |
|  | R:ACTGGTAAACCAGGCCAG |  |  |  |
| TEM | F:KACAATAACCCTGRTAAATGC | 919 | 48 | PCR |
|  | R:AGTATATATGAGTAAACTTGG |  |  |  |
| CTXM-M-1 | F:AAGACTGGGTGTGGCATTGA | 670 | 60 | PCR |
|  | R:AGGCTGGGTGAAGTAAGTGA |  |  |  |
| CTXM-M-9 | F:GCTTTATGCGCAGACGAGTG | 686 | 60 | PCR |
|  | R:GCCAGATCACCGCAATATCA |  |  |  |
| SHV | F:CTTTACTCGCCTTTATCGGC | 1031 | 62 | PCR |
|  | R:TTACCGACCGGCATCTTTCC |  |  |  |
| **Housekeeping genes** | |  |  |  |
| ropB | F: GGCGAAATGGCWGAGAACCA | 501 | 55 | PCR |
|  | R: GAGTCTTCGAAGTTGTAACC |  |  |  |
| mdh | F: CCCAACTCGCTTCAGGTTCAG | 477 | 67 | PCR |
|  | R: CCGTTTTTCCCCAGCAGCAG |  |  |  |
| pgi | F:GAGAAAAACCTGCCTGTACTGCTGGC | 432 | 60 | PCR |
|  | R:CGCGCCACGCTTTATAGCGGTTAAT |  |  |  |
| phoE | F:ACCTACCGCAACACCGACTTCTTCGG | 420 | 60 | PCR |
|  | R: TGATCAGAACTGGTAGGTGAT |  |  |  |
| infB | F: CTCGCTGCTGGACTATATTCG | 318 | 55 | PCR |
|  | R: CGCTTTCAGCTCAAGAACTTC |  |  |  |
| tonB | F:CTTTATACCTCGGTACATCAGGTT | 414 | 62 | PCR |
|  | R:ATTCGCCGGCTGRGCRGAGGAG |  |  |  |
| gapA | F: TGAAATATGACTCCACTCACGG | 450 | 60 | PCR |
|  | R:CTTCAGAAGCGGCTTTGATGGCTT |  |  |  |
| **Capsule gene** | |  |  |  |
| wzi | F:GTGCCGCGAGCGCTTTCTATCTTGGTATTCC | 580 | 56 | PCR |
|  | R:GAGAGCCACTGGTTCCAG  AA[CorT]TT[CorG]ACCG |  |  | PCR |
| rmpA | F:GAGTAGTTAATAAATCAATAGCAAT | 332 | 50 | PCR |
|  | R:CAGTAGGCATTGCAGCA |  |  |  |
| rmpA2 | F:GTGCAATAAGGATGTTACATTA | 430 | 50 | PCR |
|  | R:GGATGCCCTCCTCCTG |  |  |  |
| peg-344 | F:CTTGAAACTATCCCTCCAGTC | 508 | 53 | PCR |
|  | R:CCAGCGAAAGAATAACCCC |  |  |  |
| Aerobactin | F:GCATAGGCGGATACGAACAT | 556 | 55 | PCR |
|  | R:CACAGGGCAATTGCTTACCT |  |  |  |
| iucA | F:GCTTATTTCTCCCCAACCC | 583 | 59 | PCR |
|  | R:TCAGCCCTTTAGCGACAAG |  |  |  |
| iroN | F:AAGTCAAAGCAGGGGTTGCCCG | 668 | 55 | PCR |
|  | R:GACGCCGACATTAAGACGCAG |  |  |  |
| iroB | F:ATCTCATCATCTACCCTCCGCTC | 235 | 59 | PCR |
|  | R:GGTTCGCCGTCGTTTTCAA |  |  |  |
| YbtA | F:GACGGAAACAGCACGGTAAA | 242 | 50 | PCR |
|  | R:GAGCATAATAAGGCGAAAGA |  |  |  |
| **iutA** | F:GGGAAAGGCTTCTCTGCCAT | 920 | 63 | PCR |
|  | R:TTATTCGCCACCACGCTCTT |  |  |  |

Supplementary Table 2. Distribution of KPBSI and CRKPBSI patients across different wards in thre hospitals.

| **Wards** | **number(n)** | **percentage(%)** |
| --- | --- | --- |
| Other Wards*（Distribution of KPBSI patients across different wards in the Affiliated Hospital of Southwest Medical University） |  |  |
| Oncology Ward | 9 | 3.2% |
| Infectious Diseases Ward | 8 | 2.9% |
| Nephrology Ward | 8 | 2.9% |
| Cardiology Ward | 8 | 2.9% |
| Rehabilitation Medicine Ward | 8 | 2.9% |
| Gastrointestinal Surgery Ward | 7 | 2.5% |
| Spinal Surgery Ward | 6 | 2.2% |
| Rheumatology and Immunology Ward | 5 | 1.8% |
| Orthopedics and Joint Surgery Ward | 4 | 1.4% |
| Vascular Surgery Ward | 3 | 1.1% |
| Pain Management Ward | 2 | 0.7% |
| Obstetrics Ward | 2 | 0.7% |
| Gynecology Ward | 1 | 0.4% |
| Thyroid Surgery Ward | 1 | 0.4% |
| Geriatrics Ward | 1 | 0.4% |
| Traditional Chinese Medicine Ward | 1 | 0.4% |
| Plastic and Burn Surgery Ward | 1 | 0.4% |
|  |  |  |
| Other Wards#（Distribution of KPBSI patients across different wards in Zigong Fourth People's Hospital） |  |  |
| Oncology Ward | 4 | 3.8% |
| Neurology Ward | 4 | 3.8% |
| Gastrointestinal Surgery Ward | 3 | 2.9% |
| General Medicine Ward | 2 | 1.9% |
| Cardiothoracic Surgery Ward | 1 | 1.0% |
| Plastic and Burn Surgery Ward | 1 | 1.0% |
| Orthopedics and Joint Surgery Ward | 1 | 1.0% |
|  |  |  |
| Other Wards &（Distribution of KPBSI patients across different wards in the Second People's Hospital of Neijiang） |  |  |
| Orthopedics and Joint Surgery Ward | 1 | 2.1% |
| Rehabilitation Medicine Ward | 1 | 2.1% |
| Nephrology Ward | 1 | 2.1% |
| Emergency Medicine Department Ward | 1 | 2.1% |
|  |  |  |
| Other Wards@（ Distribution of total KPBSI patients across different wards in the three hospitals） |  |  |
| Neurosurgery Ward | 15 | 3.5% |
| Emergency Medicine Ward | 14 | 3.3% |
| Nephrology Ward | 14 | 3.3% |
| Cardiology Ward | 13 | 3.0% |
| Gastrointestinal Surgery Ward | 10 | 2.3% |
| Rehabilitation Medicine Ward | 9 | 2.1% |
| Orthopedics and Joint Surgery Ward | 6 | 1.4% |
| Spinal Surgery Ward | 6 | 1.4% |
| Rheumatology and Immunology Ward | 5 | 1.2% |
| General Medicine Ward | 4 | 0.9% |
| Cardiothoracic Surgery Ward | 3 | 0.7% |
| Vascular Surgery Ward | 3 | 0.7% |
| Obstetrics Ward | 2 | 0.5% |
| Pain Management Ward | 2 | 0.5% |
| Plastic and Burn Surgery Ward | 2 | 0.5% |
| Gynecology Ward | 1 | 0.2% |
| Thyroid Surgery Ward | 1 | 0.2% |
| Geriatrics Ward | 1 | 0.2% |
| Traditional Chinese Medicine Ward | 1 | 0.2% |
|  |  |  |
| Other Wards^（Distribution of total CRKPBSI patients across different wards in southern Sichuan, China.） | 7 | 10.6% |
| Rheumatology and Immunology Ward | 1 | 1.5% |
| Emergency Medicine Department Ward | 1 | 1.5% |
| Urology Ward | 1 | 1.5% |
| Endocrinology and Metabolism Ward | 1 | 1.5% |
| General Medicine Ward | 1 | 1.5% |
| Gastrointestinal Surgery Ward | 1 | 1.5% |
| Cardiothoracic Surgery Ward | 1 | 1.5% |
